# Practical and Computational Studies of Bivalence Metal Complexes of Sulfaclozine and Biological Studies

**DOI:** 10.3389/fchem.2021.644691

**Published:** 2021-06-15

**Authors:** Abeer A Sharfalddin, Abdul-Hamid Emwas, Mariusz Jaremko, Mostafa A. Hussien

**Affiliations:** ^1^ Department of Chemistry, Faculty of Science, King Abdulaziz University, Jeddah, Saudi Arabia; ^2^ King Abdullah University of Science and Technology (KAUST), Thuwal, Saudi Arabia; ^3^ King Abdullah University of Science and Technology (KAUST), Biological and Environmental Science and Engineering (BESE), Thuwal, Saudi Arabia; ^4^ Department of Chemistry, Faculty of Science, Port Said University, Port Said, Egypt

**Keywords:** electronic paramagnetic resonance analysis, DFT, sulfaclozine, molecular docking, anticancer

## Abstract

In the search for novel, metal-based drug complexes that may be of value as anticancer agents, five new transition metal complexes of sulfaclozine (SCZ) with Cu(II), Co(II), Ni(II), Zn(II), and Fe(II) were successfully synthesized. The chemical structure of each complex was characterized using elemental analysis (CHN), IR spectroscopy, UV–Vis spectroscopy, thermogravimetric analysis (TGA), and electronic paramagnetic resonance (EPR) spectroscopy. IR spectra indicated that the donor atoms were one sulfonyl oxygen atom and one pyrazine nitrogen atom, which associated with the metal ions to form a stable hexagonal coordination ring. The metal–ligand stability constant (K_f_) revealed that Cu(II) and Ni(II) have good coordination stability among the metal compounds. Theoretical studies using DFT/B3LYP were performed to further validate the proposed structures. The obtained results indicated that Cu(II) has a trigonal bipyramidal geometry, whereas Fe(II), Co(II), and Ni(II) have an octahedral structure, while Zn(II) has a tetrahedral arrangement. The bio-activities of the characterized complexes were evaluated using DNA binding titration and molecular docking. The binding constant values for the metal complexes were promising, with a maximum value for the copper metal ion complex, which was 9 × 10^5^ M^-1^. Molecular docking simulations were also carried out to evaluate the interaction strength and properties of the synthesized metal complexes with both DNA and selected cancer-relevant proteins. These results were supported by *in vitro* cytotoxicity assays showing that the Cu(II) and Ni(II) complexes display promising antitumor activity against colon and breast cancer cell lines.

## Introduction

Sulfonamide is a well-known antibacterial compound that has been in use for around 50 years (Stober and DeWitte, 1982). It came to prominence when Domagk et al. reported that Prontosil, a sulfamidochrysoidine azo dye, was reduced to the antibiotic sulfonamide and triamine benzene in living cells (Domagk, 1935), with sulfonamide being the affected part in this dye molecule. Metal ions have played key roles as components of pharmaceuticals in the field of anticancer therapy (Wong and Giandomenico, 1999), arthritis (Roberts et al., 1996), and cardiovascular medicine (Navarro et al., 2004). Thus, searching for novel metal-based drug complexes is a high priority for medicinal biochemists.

**FIGURE 1:**
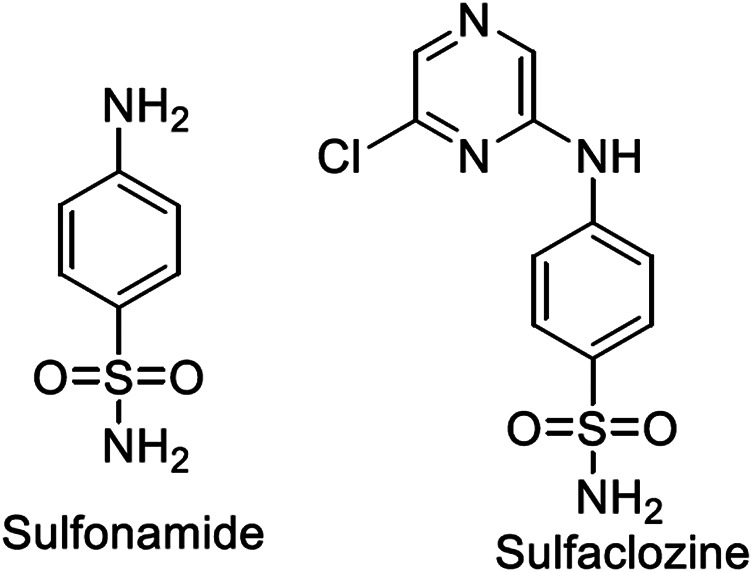
Structures of sulfonamide and sulfaclozine.

Metal complexes of sulfonamide drugs, Figure 1, have drawn attention from the scientific community because of their superior clinical applications compared to the free drugs. For instance, the zinc sulfadiazine complex has a 1:2 molar ratio and is used to promote wound healing and control infections (Fox, 1977). Additionally, the Ag(I) sulfadiazine complex is utilized as a topical antibacterial agent for treating first-, second-, and third-degree burns (Carr et al., 1973). Due to the effectiveness of sulfonamide metal complexes in the clinic, a diversity of metal complexes, metals based on sulfonamide or its derivative compounds, with transition metals, Cu(II), Co(II), and Ni(II) (Ajibade et al., 2006; Rocha et al., 2019), or with transition metals of platinum group, Pt(II), Pd(II) (Ajibade et al., 2013), and Ru(III) (Refat et al., 2016), or with heavy metals (Khedr and Saad, 2015), have been obtained to enhance their antimicrobial properties (Ajibade et al., 2006; Rocha et al., 2019).

Sulfaclozine (SCZ), Figure 1, is a derivative of the sulfonamide drug in which an amide group (NH) binds to a chloropyrazine aromatic ring. It is used as an oral antibiotic to treat various poultry diseases (Şentepe and Eraslan, 2010) and murine toxoplasmosis (Zeng et al., 2012; Ismail et al., 2016). Interestingly, it has several potential binding sites that could be coordinated with metal ions, namely, two pyrazine nitrogen atoms, sulfonyl oxygen atoms, and sulfonamide nitrogen atoms and an amino group at its tail. In addition, the chloropyrazine ring in SCZ could enhance its biological properties more than sulfonamide.

To the best of our knowledge, no published reports have described the complexation between divalent transition metals and SCZ. In this work, a new synthesis of metal complexes in ethanol solution was performed. The molecular structures of all the new complexes were characterized by analytical, spectroscopic, and thermal techniques. The structures of the prepared complexes were optimized by DFT with the exchange–correlation functional approach (B3LYP) to study the geometric arrangement around the metal ions. Moreover, the energy gap calculated from the HOMO and LUMO was used to predict the biological properties. Experimentally, various techniques were carried out to investigate the potential influence of the metal ion coordination on their potential as therapeutics. One of the least expensive and simplest methods is spectroscopic titration experiments with CT-DNA to study binding affinity of the metal complexes with the pharmacological target. A molecular docking approach was also used to examine the molecular interaction of the newly synthesized compounds and the free ligand to test their inhibitory capacity toward different cancer proteins. A previous study tested the inhibitory effect of sulfonamide and its derivatives on a selected cancer cell line (Refat et al., 2016). Hence, in this work, *in vitro* cytotoxicity assays using two cell lines, a breast cancer cell line (MCF-7) and a colon cancer cell line (CaCo-2), were used to evaluate all compounds.

## Materials and Methods

### Chemicals and Reagents

Sulfaclozine (SCZ) of 99% purity was purchased from Aldrich. The metal chlorides were obtained from Fisher Scientific. Solvents and analytical reagents were commercially obtained from the BDH chemical company and used as received.

### Synthesis of Metal Complexes

The appropriate ratio of metal chlorides of Cu(II), Co(II), Zn(II), Fe(II), and Ni(II) of 1.0 mmol volume in 20 ml ethyl alcohol was added to the SCZ ligand (2.0 mmol in 30 ml ethyl alcohol). The mixed solution was placed on a hot plate at 80°C for 2–3 h with continuous stirring and refluxing until the color precipitates formed. The resulting solution was allowed to evaporate at room temperature, and the solids were washed with diethyl ether and dried under vacuum.

### The Molar Ratio Method and the Metal Sulfaclozine Stability Constants Procedure

The stoichiometric composition of the complexes in ethanol was determined by the molar relation method (Davila et al., 2012). The concentration of the metal ion was kept constant (0.36 × 10^-4^ M), and the concentration of the SCZ ligand varied from [L] = 0.18 to 1.25 × 10^-4^ M. The solutions were stabilized at 25.0°C for 10 min to let the reaction reach equilibrium. Next, the spectra were recorded in the Shimadzu UV/Vis spectrometer with a cell width of 1 cm optical path length, in the 200–500 nm range.

The metal–ligand stability constant (K_f_) of the complexes was calculated using the following equilibrium equation:

$$\left[M\right]+2\left[\text{SCZ}\right]↔\left[ML_{2}\right],$$

$$K_{f}=\left(\frac{\left[ML_{2}\right]}{\left[M\right]\times {\left[L\right]}^{2}}\right)=\left(\frac{\left[ML_{2}\right]}{\left(C_{M}-\left[ML_{2}\right]\right)\times {\left(C_{L}-2\left[ML_{2}\right]\right)}^{2}}\right).$$

Using Beer’s law (A = ɛbc), the equation can be rewritten for the stability constants of complexes (Panhwar and Memon, 2012; Samsonowicz and Regulska, 2017) as

Kf=(Aε(CM-Aε)×(CL-2Aε)2),

where A is the measured absorbance and ɛ is the molar absorption coefficient.

### Structure Analysis

The micro-analytical ratio analyses (C, H, and N) were carried out in a PerkinElmer CHN 2400 elemental analyzer. The molar conductance of the metal complexes in the DMF solvent (10^−3^ M) was measured on a Hach conductivity meter model. A Bruker infrared spectrophotometer was utilized to record the infrared spectra of the ligand and its complex in the range of 400–4000 cm^−1^. An electronic spectroscopic study of SCZ and the metal compounds in DMSO solution with a concentration of 10^−3^ M was obtained by the Shimadzu UV/Vis spectrometer in the range of 200–1100 nm. The electron paramagnetic resonance study for the solid sample was recorded on a Bruker EMX PLUS spectrometer using the X band frequency (9.5 GHz) using reported experimental details (Alahmari et al., 2019; Alghrably et al., 2019). The content of metal ions was calculated gravimetrically as metal oxides. The TG–DTG experiment was conducted using the Mettler Toledo STARe software. All experiments were under air at a flow rate of 30 ml/min and a heating rate of 10°C/min starting from 25°C and ending at 800°C using a single loose top loop. The percentage of metal ions was calculated gravimetrically as metal oxides. Magnetic measurements of metal complexes were measured at room temperature using Gouy’s method by a magnetic susceptibility balance from the Johnson Matthey and Sherwood model.

### Computational Details

The initial molecular geometries of SCZ and the metal complexes were optimized in the gas phase using the Gaussian 09W (Frisch et al., 2009) program package employing hybrid DFT/B3LYP at 6-31G (d,p) level for the free ligand and LAND for the metal complexes. The frequencies calculated were followed by optimization to ensure that the obtained structures were in the minima energy state. The GaussView molecular visualization program (Dennington et al., 2016) was used to visualize the input files and extract the HOMO–LUMO energies. The reactivity descriptors: chemical potential (μ), global hardness (η), chemical softness (S), and electrophilicity (ω), were calculated using the following formulas (Sharfalddina et al., 2020a):

$$\text{μ}=\;-\left({\text{E}}_{\text{LUMO}}-\;{\text{E}}_{\text{HOMO}}/2\right),$$

$$\text{η}=-\left({\text{E}}_{\text{LUMO}}-{\text{E}}_{\text{HOMO}}/2\right),$$

S=1/2 η,

ω=π2/2 η R.

### Bio-Activity Analysis

#### DNA Binding Methodology

The DNA binding protocol is reported in our previous work (Alsaeedi et al., 2020; Sharfalddina et al., 2020b). Compounds were dissolved in DMSO at room temperature with a fixed concentration that had absorbances between 1 and 1.2. The CT-DNA stock solution was prepared in a buffer solution (pH = 7.4) and kept at 5°C for 1 week. The ratio absorbance for the stock at 280–290 nm was 1.8 (nucleotide to protein) indicating DNA is free of protein (Arjmand and Jamsheera, 2011). The molar absorption coefficient of 6600 M^–1^ cm^–1^ and the absorbance at 260 nm for CT-DNA were used to determine the DNA concentration (Tabassum et al., 2014; Mashat et al., 2019), which was 1.21 × 10^–4^ M. The experiments were performed by keeping the compound concentration constant and varying the DNA concentration from 1.69 × 10^-6^ to 5.55 × 10^-6^ M. The mixture solutions were allowed to incubate for 30 min at RT before recording the absorption. The binding constant was computed by the Wolfe−Shimer equation (Zehra et al., 2019) given as follows:

$$\left[\text{DNA}\right]/\left(\varepsilon_{\text{a}}-\varepsilon_{\text{f}}\right)=\left[\text{DNA}\right]/\left(\varepsilon_{\text{b}}-\varepsilon_{\text{f}}\right)+1/K_{\text{b}}\left(\varepsilon_{\text{a}}-\varepsilon_{\text{f}}\right),$$

where [DNA] = concentration of CT-DNA in base pairs.
ɛ_a_ = extinction coefficient observed for A_obs_/[compound] at the given DNA concentration.
ɛ_f_ = extinction coefficient of the free compound in solution.
ɛ_b_ = extinction coefficient of the compound when binding to DNA.
K_b_ = ratio of the slope to the intercept of the plot [DNA]/(є_a_–є_f_) versus [DNA].
K_b_ values were obtained by plotting the left side of the equation vs. DNA concentration and then calculating the ratio of the slope and intercept. The following equation was used to calculate Gibb’s free energy values:
ΔG = −RT ln K_b_, where R = 8.314 JK^-1^ mol^-1^ and T = 298K.

#### Molecular Docking

High-resolution crystallographic structures of proteins included in this study, breast cancer (PDB code = 1hK7) and colon cancer (PDB code = 4FM9) receptors, were downloaded from the Protein Data Bank into MOE software 2015 (MOE (The Molecular Operating Environment), 2015). The docking protocol is reported in our previous work (Abdel-Rhman et al., 2019). Protein preparation started with removing water molecules and co-ligand. The site finder was used to find the possible binding sites in the protein, and then the 3D protonation process was carried out to correct and fix the protein. The 3D structures of the compounds were minimized through the MMFF94X Force Field and optimized to obtain the lowest energy conformation with the best geometry using a gradient of 0.001. The docking parameters were the triangle matcher method for placing the compound and London dG for scoring and GBVI/WSA dG for rescoring. The ranking affinity of the ligand and metal compounds toward the amide protein was calculated using binding free energy and hydrogen bonds between the ligand and the amino acid. The measured hydrogen bond did not exceed the length of 3.1–3.7 A^°^.

#### Antitumor Assay

A human colon cancer cell line (CaCo-2) and human breast cancer cell line (MCF-7) were obtained from the VACSERA Tissue Culture Collection Unit. The propagation was done in Dulbecco’s modified Eagle’s medium (DMEM) completed with heat-inactivated fetal bovine serum (10%), 1% HEPES buffer, L-glutamine, and gentamicin (50°µg/ml). Next, in a humidified atmosphere with 5% carbon dioxide, the cells were kept at 37°C and were sub-cultured two times a week. The determination of sample cytotoxicity on cells (MTT protocol) was performed as reported (Alley et al., 1988; Van de Loosdrecht et al., 1994). 1 × 10^5^ cells/ml (100 µl/well) were incubated in a 96-well tissue culture plate at 37°C for 24 h to create a complete monolayer sheet. After an aggregate sheet of cells was formed, the monolayer cells were separated from the growth medium and washed twice with wash media. 2% of serum as a maintenance medium was used to dilute the tested sample twofold in the RPMI medium. A constant volume (0.1 ml) of each diluted sample was added simultaneously in various wells in the maintenance medium at 37°C, with three wells without treatment used as control cells. The samples were checked for any physical signs of toxicity such as partial or complete loss of the monolayer every 24 h. The MTT protocol depends on tetrazolium salt reduction from yellow to purple by metabolically viable cells. Therefore, 20 µl of the solution (5 mg/ml in PBS) was added to each well and maintained (37°C, 5% CO_2_) for 1–5 h until the cell metabolization process was completed. After drying the wells by dumping the media, 200 µl DMSO was added to re-suspend the MTT metabolic product and was mixed thoroughly. The spectrophotometric absorbance at OD = 560 nm was measured and then subtracted from the background sample (50 µL MTT + 50 µL of media) at 620 nm.

The percentage of cell survival was calculated as follows:

$$\mathrm{Survival fraction=}\frac{O\mathrm{.D}.\left(\mathrm{treated cells}\right)}{O\mathrm{.D}.\left(\mathrm{controlled cells}\right)}\times 100.$$

Each experiment was repeated three times to obtain a linear relationship between optical density and cell quantity.

## Results and discussion

### Analytical Data of the Metal Complexes

The analytical data and physical properties of the ligand and its metal complexes are summarized in Table 1. The isolated colored solid complexes are stable at room temperature, except for the Zn(II) complex, which turned light brown due to the absorption of water molecules over time. Moreover, they are soluble in DMF and DMSO. The molar conductance of Cu(II) and Co(II) at 10^–3^ M in DMF had values fall in the 66–95 Ώ cm^2^ mol^-1^ range, indicating the presence of two ion types in the solution, which are 1:1 of cationic and anionic species (Ali et al., 2013). The zinc complex showed a higher value, 152 Ώ cm^2^ mol^-1^, suggesting two Cl^–^ ions out of the coordination sphere. In contrast, for Ni(II) and Fe(II), the molecular conductance was too low to account for any dissociation to Cl^–^ ions; thus, they are non-electrolytes.

**TABLE 1:** Analytical and physical data of SCZ and metal complexes.

Metal complex	M.Wt.	Color	Elemental analysis, % found (calc.)				Λ_m_ (Ώcm^2^ mol^-1^)	Melting point
			C%	N%	S%	M%				
SCZ	250.05	White	47.99	22.39	12.31	-	1.3	130
			48	22.40	12.30			
[Cu(SCZ)_2_Cl]Cl	635	Yellow ochre	34.13	15.92	9.10	9.09	94	170
			34.10	15.95	9.12	9.03		
[Co(SCZ)_2_ClOH_2_]Cl	628.39	Blue	34.35	15.89	10.17	9.35	70	205
			34.32	15.89	10.20	8.50		
[Ni(SCZ)_2_Cl_2_]	630.15	Light green	34.36	15.99	9.17	8.40	5.56	200
			34.40	16.05	9.19	8.41		
[Fe(SCZ)_2_Cl_2_]	627.30	Dark brown	34.52	16.10	9.21	8.02	1.56	158
			34.51	15.92	9.25	8.00		
[Zn(SCZ)_2_]Cl_2_	636.83	Sandy	34.04	15.60	9.09	9.26	152	214
			34.02	15.55	9.10	9.30		

### Stoichiometry and Metal–Ligand Stability Constant

The collected absorption is plotted toward the ratio of [M]/[M]+[L] and presented in Supplementary Figure S1 for the metallic complexes. The reflection line upon increasing the ligand concentration was around 0.33 and revealed that one mole of the metal reacted with two moles of the ligand.

The obtained values of K_f_ by the previous equations were in the order Zn−SCZ (1.74 × 10^-5^ ) >Cu−SCZ (1.47 × 10^-5^ ) > Ni−SCZ (0.48 × 10^-5^ ) > Fe–SCZ (0.25 × 10^-5^ ) > Co−SCZ (0.17 × 10^-5^) and showed good interaction of Zn(II) and Cu(II) ions forming a stable coordination complex.

### Infrared Spectroscopy

The comparison spectra of the free ligand and the five metal complexes are illustrated in Supplementary Figure S2, and the essential bands are given in Supplementary Table S1. The divalent metal complexes had similar infrared spectra to their SCZ drug, and thus, careful observation of peak shift was performed. The NH_2_ stretching appeared at 3295–2966 cm^-1^ for asymmetric and symmetric modes, respectively, maintained in the same range for all complexes. As a consequence of the hydrogen bonding interaction between the NH_2_ and SO_2_ groups, a significant shift to higher frequencies (Rocha et al., 2019) was observed for those bands. Moreover, the NH_2_ binding at 1682 cm^-1^ was preserved in the metal spectra, which revealed this assignment is not coordinated to the metal. Another donating atom group is the oxygen atoms of the SO_2_ group, which could be associated with the metal center. There is noticeable disappearing for the symmetric SO_2_ at 1149 cm^−1^ or red-shift for asymmetric stretching modes at 1344 cm^−1^ indicating the coordination of the sulfonamide oxygen to the metal ion. The intensity bands corresponding to the C = N group in the pyrazine ring at 1580,1512, and 1433 cm^-1^(Stober and DeWitte, 1982) shifted slightly after coordination to the metal concerning those of the free ligand, thus proving that N_4_ pyridine associated with the complexation to form a hexagon ring. The assignment of the M–O and M–N stretching modes in the metal complex spectra was indicated by the low-intensity band in the ranges 742–600 cm^-1^ and 420–400 cm^-1^, respectively.

### Electronic Paramagnetic Resonance Analysis for the Cu(II) Complex

**FIGURE 2:**
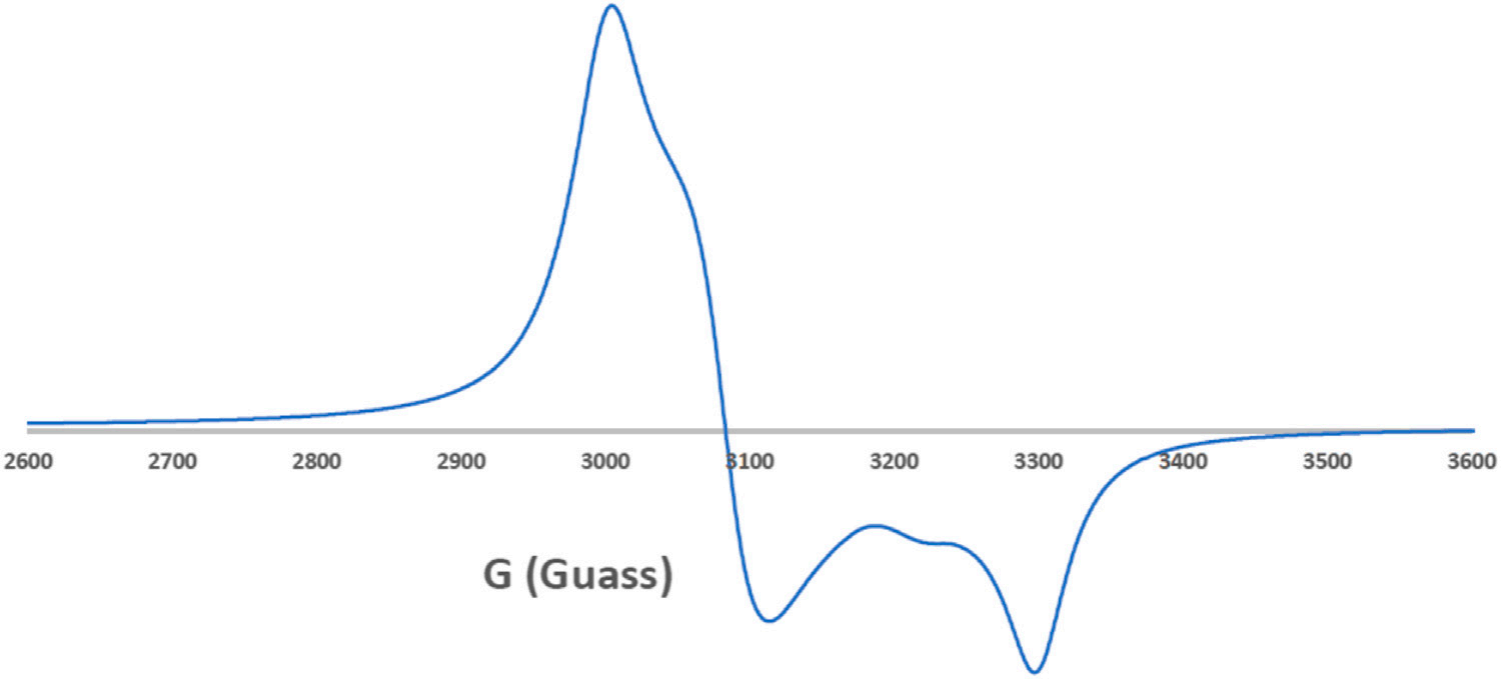
EPR spectrum of the Cu–SCZ complex.

EPR spectroscopy is a selective method where only unpaired electron species can be detected, while all other parts of the studied molecules are EPR silent. Thus, EPR spectroscopy is a powerful approach to study the formation of organic radicals (Mattar et al., 2002) and monitor the coordination of paramagnetic transition metals such as the Cu(II) and Mn(II) complexes (Emwas et al., 2013; Haque et al., 2019). In this study, we employed EPR spectroscopy to study the ligand coordination of the [Cu(SCZ)_2_Cl]Cl complex. The solid EPR presented in Figure 2 shows two peaks with different g-values: the one with parallel orientation with term gǁ and the other with perpendicular orientation with term g⊥, which was higher than the last one (g⊥ = 2.189 > g‖ = 2.044). This value suggested a compression on the Z axial and a pentacoordinate arrangement strongly shifted toward the trigonal bipyramid (Kozlevčar, 2008), with a Cl^-^ atom and two oxygen atoms from two different ligand molecules in the equatorial plane and two nitrogen atoms in the axial position. The ground state will be ^2^A_1g_, which is a combination of d*z* ^2^ and d*x* ^2^–*y* ^2^ orbitals (Garribba and Micera, 2006; Lakshmi et al., 2012). Moreover, the nature of binding to the SCZ ligand was calculated by g_av_ = (g‖ + 2 g_┴_)/3 (Ibrahim et al., 2015) and was 2.14< 2.3, which indicated a highly covalent character of the metal–ligand bond.

### Electronic Spectroscopic and Magnetic Susceptibility

The UV–Vis spectroscopic analysis was performed for the ligand and the metal complexes in DMSO in the range of 200–900 nm, Supplementary Figure S3. The bands at 276 nm of both ligand and complex spectra can be assigned to an n→π transition. Moreover, a band between 300 and 400 nm was observed in the free ligand for the π→π* transition of the aromatic ring (Zhao et al., 1992) and shifted to a higher frequency in all metal complexes, confirming the coordination to the aromatic pyrazine ring (Yang et al., 2005). There is a peak at low energy in the range 10, 500–14, 600 cm^-1^ in the Cu(II) spectrum, suggesting the symmetry of D_3h_ for the five coordination Cu(II) complexes (Slade et al., 1968). The observed absorption at 790 nm was assigned to the allowed transition state ^2^A_1_→ ^2^E (Slade et al., 1968; Sabolová et al., 2011). The complex [Ni(SCZ)_2_]Cl_2_ showed an octahedral structure, indicated by the magnetic moment of 2.63 BM. Furthermore, two bands at 673 and 755 nm were assigned to the transition states ^3^T_1_ → ^3^A_2_ and ^3^T_1_ (F) → ^3^T_1_ (P) (Ramírez-Delgado et al., 2015), respectively. The spectrum of the cobalt compound has essential transitions bands for the octahedral structure that were from ^4^T_1g →_^4^T_2g_ and from ^4^A_2g_ → ^4^T_1g_ located at 605 and 685 nm, respectively. As reported, the octahedral structure of Fe(II) can be confirmed by the absence of the band around 1100 nm (Goodwin, 1976; Gütlich et al., 1996), Supplementary Figure S3. The brown color is due to charge transfer transitions from the metal to the ligand orbitals (Gütlich et al., 1996). Moreover, the magnetic moment for Fe(II) confirmed the low spin d^6^ configuration (0.952 BM). The nephelauxetic parameters such as the interelectronic repulsion parameter (B), covalency factor (β), and ligand-field splitting energy (10Dq) are listed in Table 2, calculated by the following equations (James E. House, 2013; König, 1971) for Co(II):

$$\text{Dq}=\left[{\left(85\text{V}32\;-\;4\left(\text{V}3-2\text{V}2\right)2\right)}^{\text{½}}-9\left(\text{V}3-2\text{V}2\right)\right]/340,$$

B=(V3−2V2+30Dq)/15,

β=B/B0(B0 = 971),

while for Ni(II),

$$\text{Dq}=\left[\left(9\text{V}2+\text{ V}3\right)-{\left(85\left(\text{V}2-\text{V}3\right)2-4\left(\text{V}2+\text{V}3\right)2\right)}^{\text{½}}\right]/340,$$

B=(V2+V3−30Dq)/15,

β = B/B0 (B0 =1030).

**TABLE 2:** Electronic parameters of the metal complexes.

Compound	V_ligand band shift_		V3	V2	10Dq	B	β
SCZ	274(36500)	313(31500)		-	-		
[Cu(SCZ)_2_Cl]Cl	275(36400)	321(31200)		810(12346)	12350	-	-
[Co(SCZ)_2_ClOH_2_]Cl	278(36000)	315(31500)	589(16977)	685(14600)	7770	708	0.730
[Ni(SCZ)_2_Cl_2_]	297(25189)	405(24700)	680(14700)	760(12900)	5400	760	0.738
[Fe(SCZ)_2_Cl_2_]	276	335(29900)	-	-	-	-	-

### Thermal Gravimetric Analysis and Kinetic Thermodynamics

**FIGURE 3:**
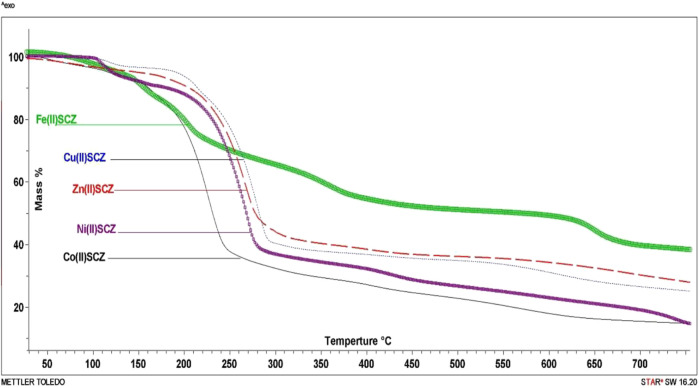
TG curves of [Cu(SCZ)_2_Cl]Cl.2H_2_O, [Co(SCZ)_2_ClOH_2_]Cl.2H_2_O, [Ni(SCZ)_2_Cl_2_].2H_2_O, [Zn(SCZ)_2_]Cl_2_.2H_2_O, and [Fe(SCZ)_2_Cl_2_].2H_2_O complexes.

The isolated solid metal complexes were analyzed by TGA to characterize the thermal stability within the temperature range of 25–800°C. The decomposed stages and their assignments are listed in Table 3. Figure 3 shows the TG curve for the metal complexes. The Cu(II) complex presented two stages starting from 150°C with the loss of two water molecules, losing weight of 4.5%. The second step was at 205–240°C with the loss of all the organic molecules (75.3 %) and leaving CuO as a final metallic residue. In addition, Co(II) and Ni(II) had the same number of water molecules in the first stage in the range 100–170°C, followed by decomposing at 250°C for anhydrase [Co(SCZ)_2_ClOH_2_]Cl and 230°C for [Ni(SCZ)_2_Cl_2_] leaving a metallic residue percentage of 17.4% and 23.2%, respectively. On the contrary, [Fe(SCZ)_2_Cl_2_].3H_2_O had four steps, which were assigned to slow degradation beginning with evaporating three outside lattice water molecules. The second step had the highest weight loss (22.5%) of the complex at 160–170°C. The last two steps were similar by losing sulfonamide and coupling amine groups at each step until 230°C to complete decomposition. The one-step Zn(II) complex was thermally stable until 200°C and began a full fragmentation that was finished at 260°C. The final resultant residues were metal oxides and counted carbon atoms.

**TABLE 3:** Thermogravimetric data of the five metal complexes.

Complex	Step	Temp. range	Weight loss % found (calc.)	Assignments	Total mass loss/% found (calc.)	Final solid state residue found (calc.)
[Cu(SCZ)_2_Cl]Cl.2H_2_O	1st	150–160	4.5 (5)	2H_2_O	79.8 (79.9)	CuO 11.3 (11.3)
	2nd	205–240	75.3 (74.7)	2HCl+2SO_2_+4N_2_+7C_2_H_4_		
[Co(SCZ)_2_ClOH_2_]Cl.2H_2_O	1st	164–172	10.1 (10.5)	2H_2_O+C_2_H_2_+2NH_3_	75.3 (76.1)	CoO+10C 17.4 (18.5)
	2nd	215–246	65.2 (65.6)	2HCl+2SO_2_+3N_2_+4C_2_H_2_		
[Zn(SCZ)_2_]Cl_2_	One step	217–260	72.7 (71.9)	2HCl+2HSO_2_+4N_2_+4C_2_H_4_	72.7 (71.9)	ZnO+7C 20.7 (20.1)
[Fe(SCZ)_2_Cl_2_].3H_2_O	1st	115–131	8.9 (7.8)	3 H_2_O	59.39 (58.9)	Fe_2_O_3_+8C 26.49 (27.83)
	2nd	157–177	22.5 (23)	2CN+5C_2_H_2_		
	3rd	302.33–340	13.4 (13.1)	2N_2_+SO_2_		
	4th	625–660	14.59 (15)	2N_2_+SO_2_		
[Ni(SCZ)_2_Cl_2_].2H_2_O	1st	80–94	10.8 (10.5)	2H_2_O+2C_2_H_2_	73.7 (74.4)	NiO+8C 23.2 (23.5)
	2nd	199–234	62.9 (63.9)	2HCl+2SO_2_+4N_2_+4C_2_H_2_		

Thermodynamic data, activation energy (Ea, kJmol^−1^), and Arrhenius factor (A, S^-1^) for the metal complexes were obtained by plotting the relationship of the Coats–Redfern (CR) equation (Coats and Redfern, 1964) or Horowitz–Metzger (HM) equation (Horowitz and Metzger, 1963), as recorded in Supplementary Table S2. The following equations were employed to calculate enthalpy activation ΔH = Ea–RT, activation entropy ΔS = R[In(Ah/kT)], and Gibbs free energy ΔG= ΔH – TΔS for each decomposition step. From the results in Supplementary Table S3, it can be seen that the activation energy E_a_ values were high and varied between 10^5^ and 10^4^ kJ mol^−1^, which translate to good stability for SCZ metal complexes. Moreover, the negative entropy values indicated the activated metal complexes need a higher-order system than the reactants. Enthalpy results support the endothermic DTG curve reactions (ΔH > 0).

### Structural Interpretation

**FIGURE 4:**
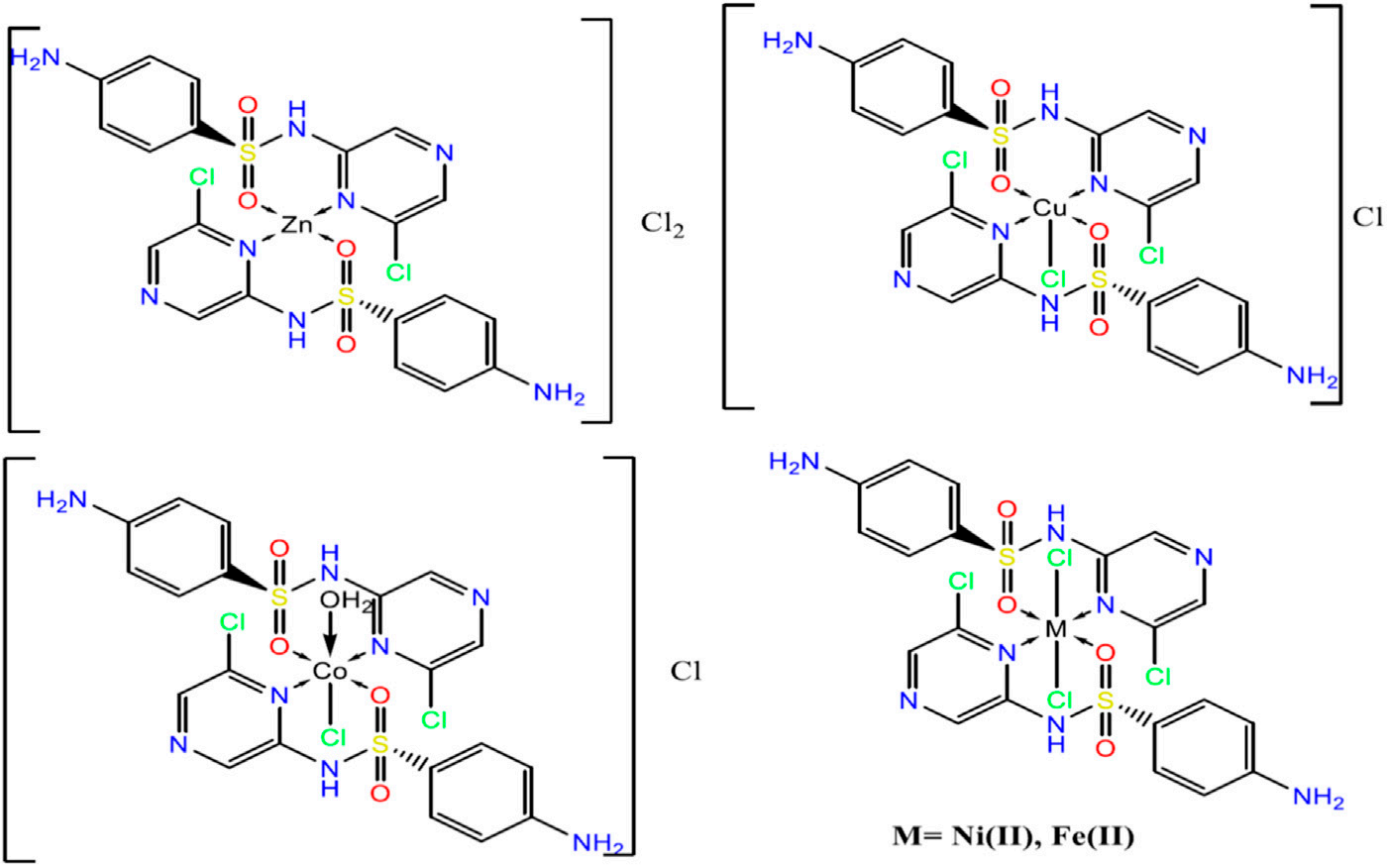
The suggested structures of the SCZ metal complexes.

From the above observations, the suggested coordination mode of the SCZ drug toward M(II) metal ions conformed with the structure and formulas designed as shown in Figure 4. The coordination sites with SO_2_ and/or pyrazine were reported in the literature (Khedr and Saad, 2015; Radha et al., 2016; Abdel-Kader et al., 2019). Moreover, the Cu(II) complex has a trigonal bipyramidal structure containing the bidentate ligand and one Cl ion. The octahedral arrangements were observed in three metal ions, Ni(II), Fe(II), and Co(II), binding to Cl ions, compatible with the conductance results. The Zn(II) complex showed a tetrahedral geometry, which is one of the passable possible structures for an ion metal (Dudev and Lim, 2000; Jana et al., 2017). There were many attempts to get single crystals from the metal complexes using the diffusion method with DMS and chloroform or ethanol and benzene but all failed. Therefore, a theoretical calculation was carried out to verify the structural and biological features.

### Molecular Orbital Calculations

#### Geometry of the Ligand and Metal Complex

**FIGURE 5:**
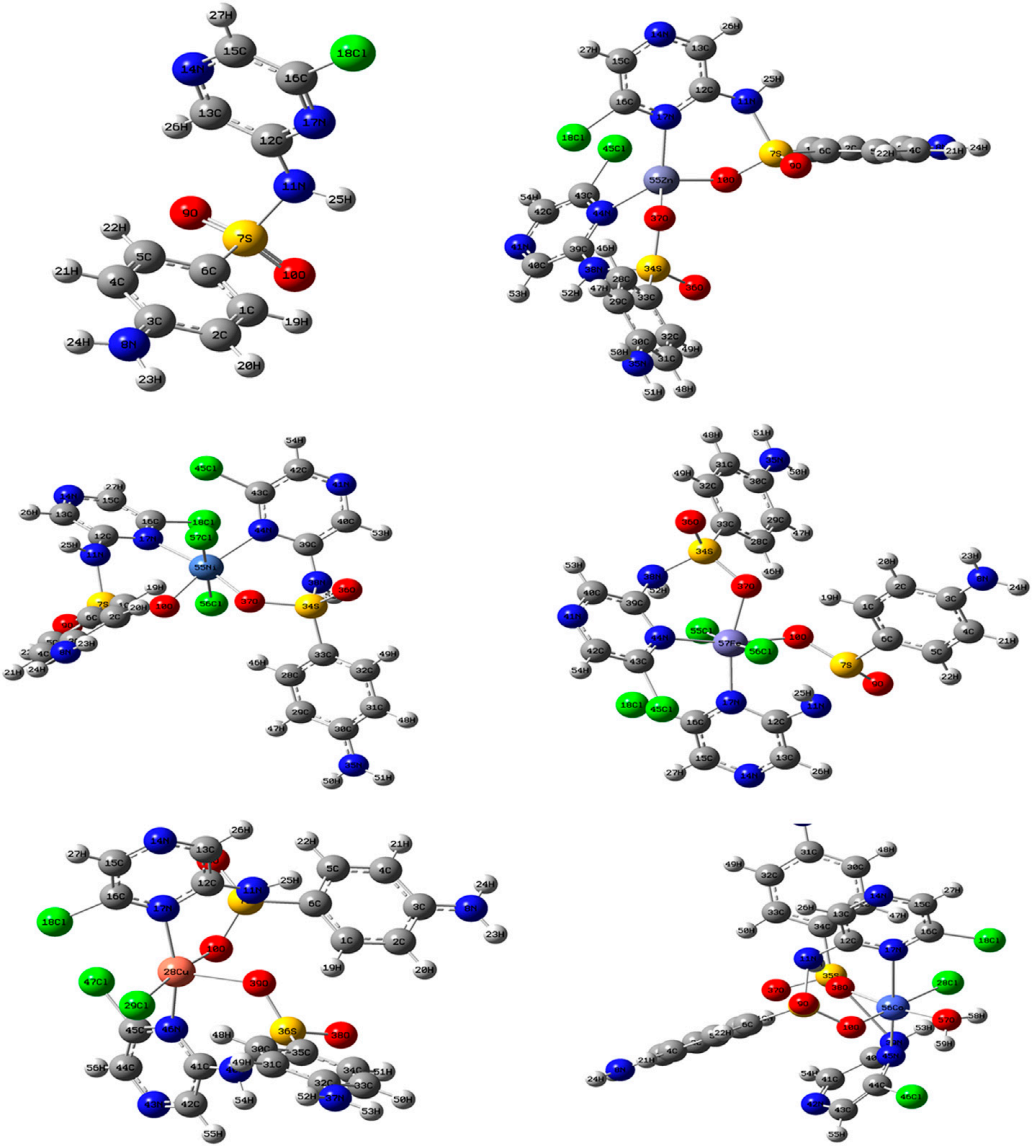
The optimized geometry with the numbering system of the free SCZ ligand and the five metal complexes.

The comparison between the optimized geometry parameters such as the bond length and the angles of the free ligand and the complexes, Cu–SCZ, Co–SCZ, Ni–SCZ, Zn–SCZ, and Fe–SCZ, is illustrated in Supplementary Table S4. The optimized geometry and numbering system of all studied metal complexes are presented in Figure 5. In general, the bond lengths around the metal ion in the complexes are longer than those of the free ligand due to the influence of the coordination process. In the Co–SCZ complex, the coordination sphere was completed with one Cl ion and one water molecule forming an octahedral arrangement. In contrast, Ni(II) and Fe(II) bonded to two Cl ions. The bond lengths of the Ni(II), Co(II), and Fe(II) ions with the donating sites of the ligand (Supplementary Table S4) suggest a minor distorted octahedral geometry around the central metal (El-Sonbati et al., 2016; Abdel-Kader et al., 2019). The new bond length of M–N and M–O bonds showed varied elongation upon complexation. Those bonds were in the range 1.94–2.4 Å, which indicates small ionic properties of the covalent bonds (Abdel-Kader et al., 2019). For the trigonal bipyramid, Cu(II) complex, the angles of O10–M–N17 and O38–M–Cl28 were 92.6 and 91.9º, respectively, which showed a small deviation from the regular penta-coordination geometry angle between the two nearest neighbor atoms (Gillespie, 1961). The average of the angles of the Zn(II) complex is 112.3º, indicating that this complex adopts a square planar with distortion by 0.03^°^.

#### The Frontier Molecular Orbital and Reactivity Properties

**FIGURE 6:**
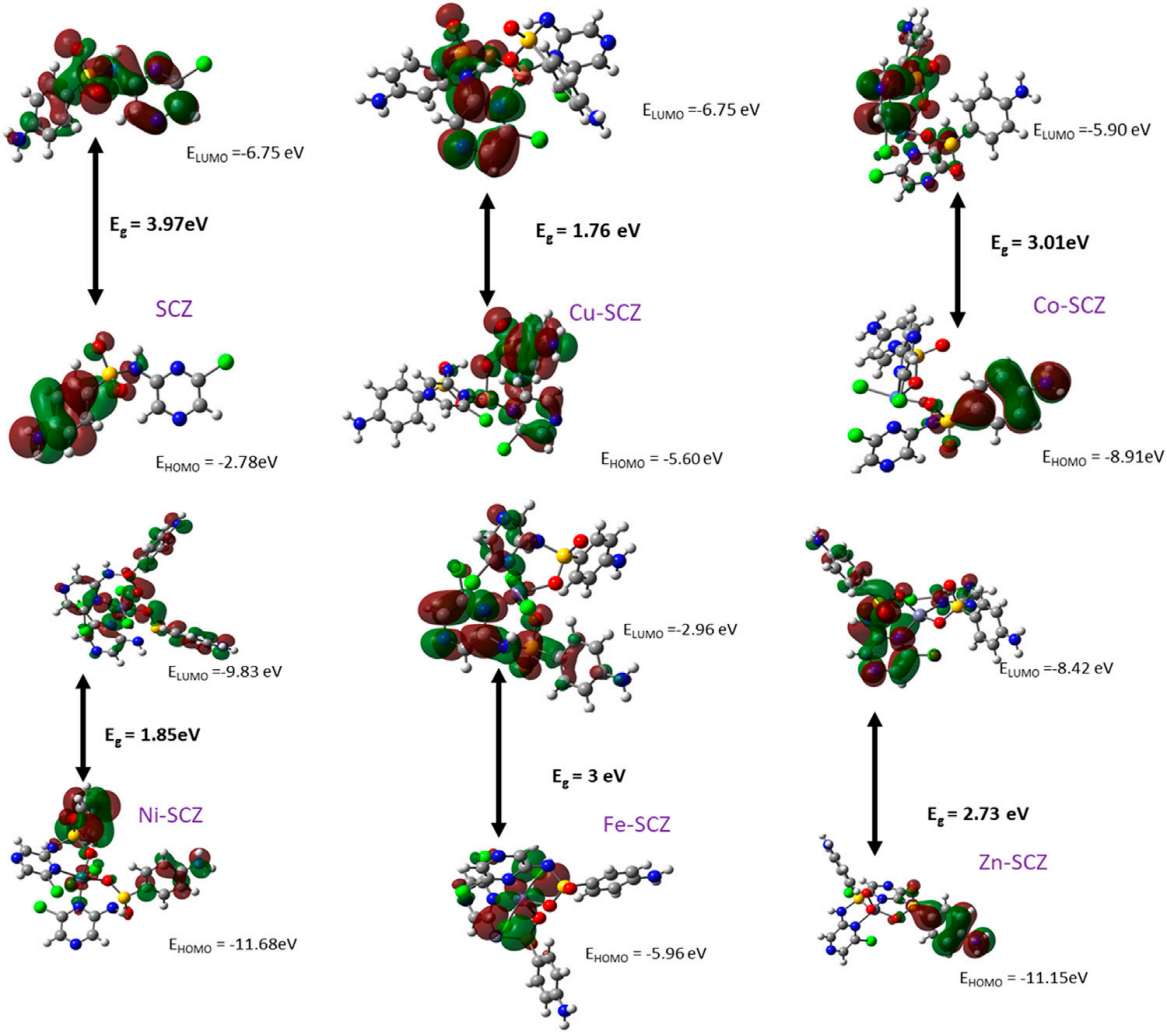
HOMO and LUMO plots of the SCZ and the metal complexes using DFT/B3LYP.

Frontier molecular orbital, FMO, studies provide the electronic characteristics of molecular systems and the reactivity of the compounds (Abdel-Kader et al., 2019; Sharfalddina et al., 2020a). Thus, the map of HOMO and LUMO energies of the studied ligand and its complexes in the ground state was extracted and is presented in Figure 6. Moreover, the calculation of the gap energy (E_g_) with the difference between the E_HOMO_ and the E_LUMO_ gives a good indicator of the molecular stability and can be used to describe the compound hardness or softness. Large E_g_ values indicate a hard molecule and low reactivity, while soft molecules have a small E_g_ value and more polarizable ability. The E_HOMO_ for the free SCZ was –3.97 eV and located on the sulfonylaniline moiety, and the E_LUMO_ = –6.75 eV was distributed over the molecule. Supplementary Table S5 presents the E_g_ and the global reactivity descriptors. The Cu(II) complex had practical reactivity among the synthesized metal complexes. Ni(II) followed this compound with low E_g_ = 1.85 eV. The chemical hardness values showed that Co(II) and Fe(II) are the most stable complexes with less reactivity. In contrast, Cu(II) and Ni(II) had the lowest values reflecting the softness and the inhibition ability of the molecules. The negative values of the chemical potential of all complexes indicated that all coordination processes are spontaneous (Rahmouni et al., 2019).

### Biological Studies

#### DNA Binding Study and Cleavage Experiments

**FIGURE 7:**
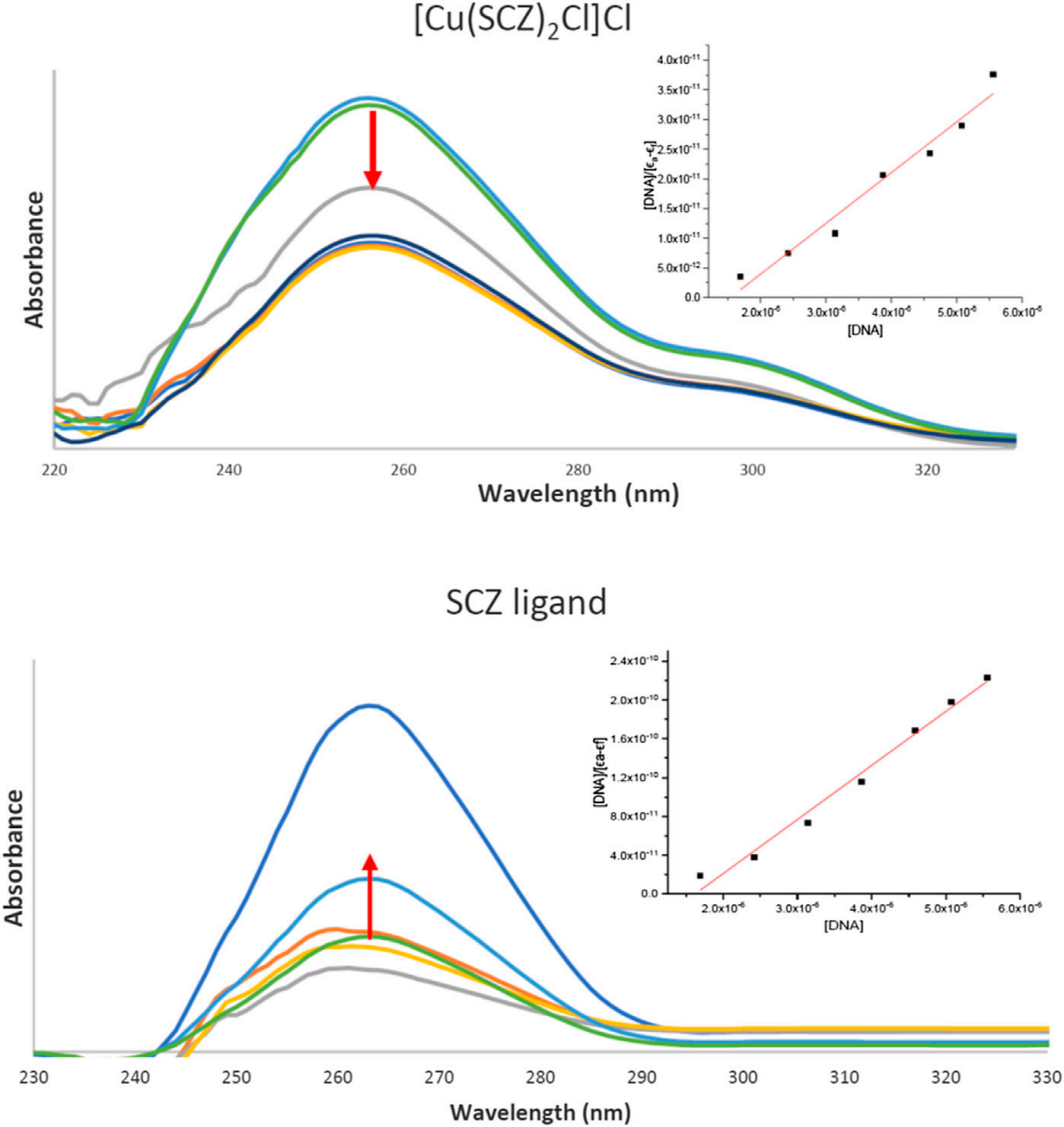
UV absorption spectra of the [Cu(SCZ)_2_Cl_2_] complex **(A)** and the ligand SCZ **(B)** in a buffer upon the addition of CT-DNA. Plots of [DNA] vs. [DNA]/ϵ_a_-ϵ_f_ for the titration of CT-DNA.

Drugs work largely by binding to a biological target such as DNA or specific protein and modifying its structure or inhibiting its activity. DNA in the biomolecules represents a major target in the development strategies of the drugs designed. Thus, a spectroscopic technique was used to study the binding ability of the free ligand and the five metal complexes simultaneously with varying concentrations of CT-DNA. In Supplementary Table S6, the maxima absorption band for the fixed concentration solution in DMSO/buffer and the binding parameter for SCZ and metal compounds are presented. Although all compounds had a blue shift, the SCZ ligand was hyperchromic in the molar absorptivity, while the SCZ metal complexes had a hypochromic effect (Figure 7A,B) due to a strong change in DNA conformation in its structure after interacting with the ligand or the metal complex (Sirajuddin et al., 2013). The hyperchromic effect is a result of DNA helix denaturation due to the resulting binding to the compound. This resulted in a limitation of the hydrogen bond between the complementary bases in the DNA double helix and the formation of a single-stranded DNA. The presence of numerous bases in free form in the solution increased the absorbance of the single-stranded DNA (Sirajuddin et al., 2013; Alsaeedi et al., 2020). Hypochromic type is due to the strong damage of the double-helical structure (Sirajuddin et al., 2012), which leads the π* orbital of the ligand in the synthesized metal complexes to couple with the π orbitals in the DNA base pairs after binding. The resulting coupled π* will be partially filled, thus decreasing the possibility of electron transition, causing hypochromicity (Ju et al., 2011; Sirajuddin et al., 2012).

The binding constant, K_b_, was between the value of the DNA minor groove binding Ru(II) complexes and classical intercalator (10^4^–10^7^ M^−1^) (Vahdati et al., 2014), thus indicating an intercalation interaction mode with the DNA biomolecule. The K_b_ value of the Cu(II) complex was higher than that of the free ligand and the other complexes, which suggested a good impact and proved the role of the cation Cu(II) in the binding process (Al-Amiery et al., 2012; Emwas et al., 2013). In sum, metal complexes could be ordered according to the decreasing Ka value: Cu(II) > Ni(II) = Fe(II) > Zn(II) > Co(II). Moreover, the negative value of ΔG showed the spontaneous interaction of the compound with DNA, Supplementary Table S6.

#### Molecular Docking

**FIGURE 8:**
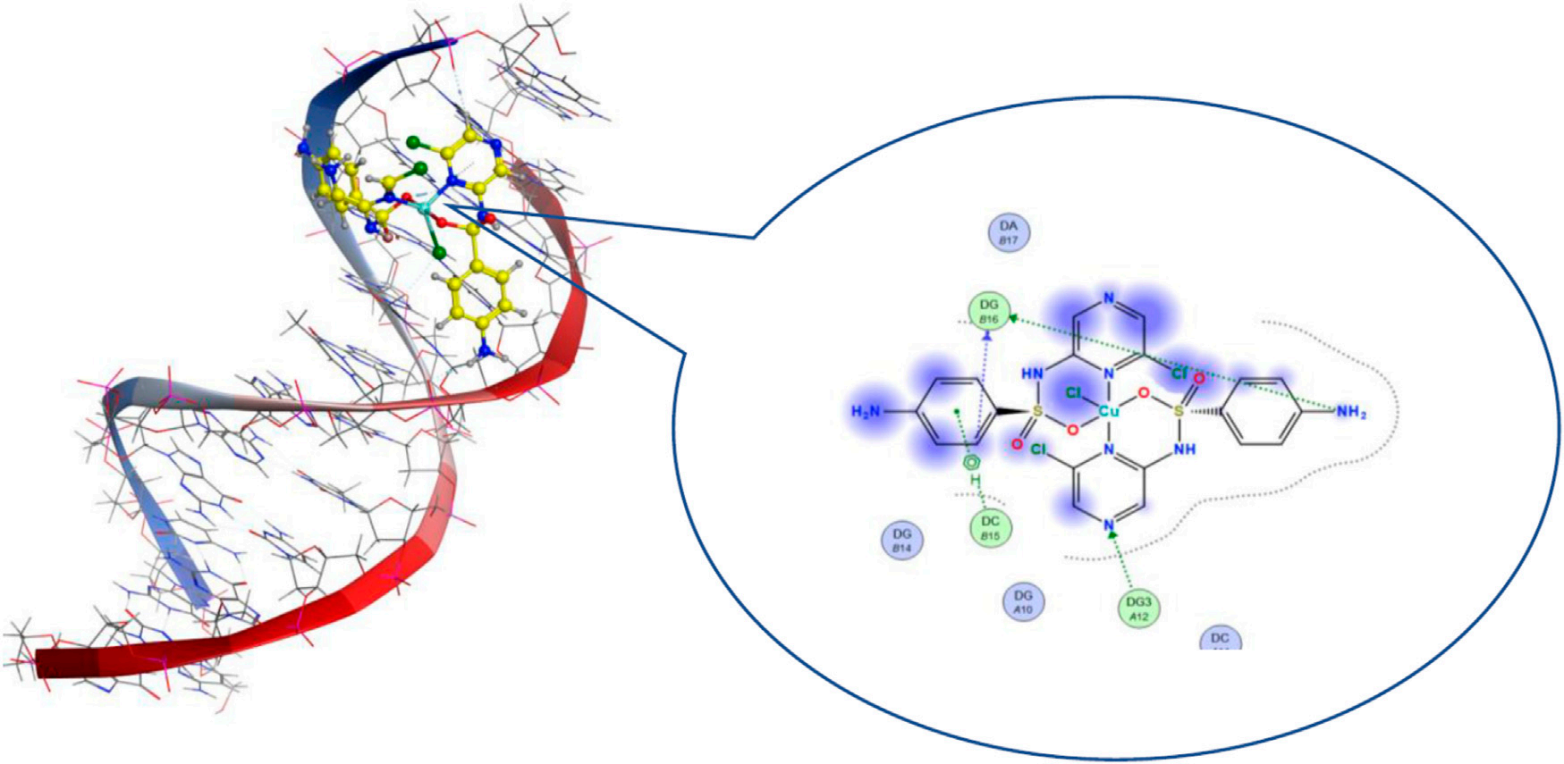
3D and 2D views of interactions of 8 with B-DNA.

Molecular docking is a theoretical calculation approach in drug design and discovery that can also be used to help scientists propose a drug interaction model and to understand the behavior of the new drug toward a biological target (Gupta et al., 2018; Márquez et al., 2020). Moreover, this method can be used to predict the binding affinity between a selected biological target and drug compounds. We performed a molecular docking study of the five metal complexes first with DNA. This revealed a strong DNA binding constant for only the Cu(II) compound. Thus, docking with a DNA helix was conducted to obtain more details of this interaction. Figure 8 shows that the Cu–SCZ complex fits well between base pairs of B-DNA, forming intercalation interactions using hydrogen bonds with amino bases leading to uncoiling of the base pairs. It has been reported that this stacking model leads to inhibition of DNA replication in rapidly growing cancer cells (Shahabadi et al., 2017).

We also performed molecular docking of the free ligand and the five metal complexes with colon and breast cancer–related proteins. For colon cancer, we selected TNIK (PDB = 2X7F) and topoisomerase II enzyme (PDB = 4F9M), which are candidate therapeutic targets for colorectal cancer (Sapna Rani and Kumar, 2014; Lee et al., 2017; Rosita and Begum, 2020). Moreover, the breast cancer–associated estrogen receptor (ID: 3ERT) and Hsp90 protein receptor (ID: 1H7K) were chosen based on previous research suggesting their value as targets for potential breast cancer therapy (Zagouri et al., 2013; Acharya et al., 2019). Table 4 presents the binding scores for the ligand and metal complexes against the selected proteins. The strongly negative values of free binding energy (S) suggest a good binding to both proteins. Generally, Cu–SCZ showed a stronger interaction pattern toward the investigated proteins than the free SCZ ligand and the other complexes. Comparing the interaction of the free ligand to the colon cancer–associated protein 2X7F with that of the Cu(II) compound, the interaction for the SCZ molecule was established by donating a hydrogen atom to the oxygen atom in glutamic acid and accepting an H bond from cysteine to one of the sulfonyl oxygen atoms. Moreover, the chloropyrazine ring interacted with both valine 39 and valine 170 to increase the free energy binding. Although the Cu(II) complex had the same binding constant with the investigated protein as the free ligand, the former had two ionic interactions with the glutamic residue with the binding energy of –6.5 kcal mol^-1^ that could enhance the interaction. In contrast, the breast cancer–associated protein receptor 3ERT displayed different binding characteristics with the free ligand and the Cu(II) complex. The amino group (NH_2_) in the SCZ molecule formed a hydrogen bond by donating this hydrogen to the glutamic and leucine oxygen atoms. The presence of two amino groups in the Cu(II) molecules elevated the interaction energies to 4.5 kcal mol^-1^ which bonded to methionine 538, methionine 343, and cysteine. Additionally, it formed an ionic interaction with the asparagine amino residue, which stabilizes this interaction more than the SCZ ligand. The different interaction models of the SCZ ligand and Cu(II) complex are presented in Table 5.

**TABLE 4:** Energy score (kcal mol^-1^) calculation for SCZ and its metal complexes toward four protein receptors.

Protein/complex		SCZ	Cu–SCZ	Co–SCZ	Ni–SCZ	Fe–SCZ	Zn–SCZ
Colon cancer protein	2X7F	–5.67657	–6.67613	–6.39108	–5.68732	–6.39108	–6.36569
	4F9M	–6.55138	–7.24939	–6.71789	–6.88243	–6.80609	–7.2411
Breast cancer protein	3ERT	–6.02021	–6.5352	–6.18126	–6.0157	–6.24734	–6.01604
	1H7K	–5.49007	–7.73247	–6.22617	–6.80484	–6.55553	–7.10101

**TABLE 5:** 2D and 3D molecular docking mode and interaction between SCZ and the Cu(II) complex with the colon protein receptor (2X7F) and the breast protein receptor (3ERT).

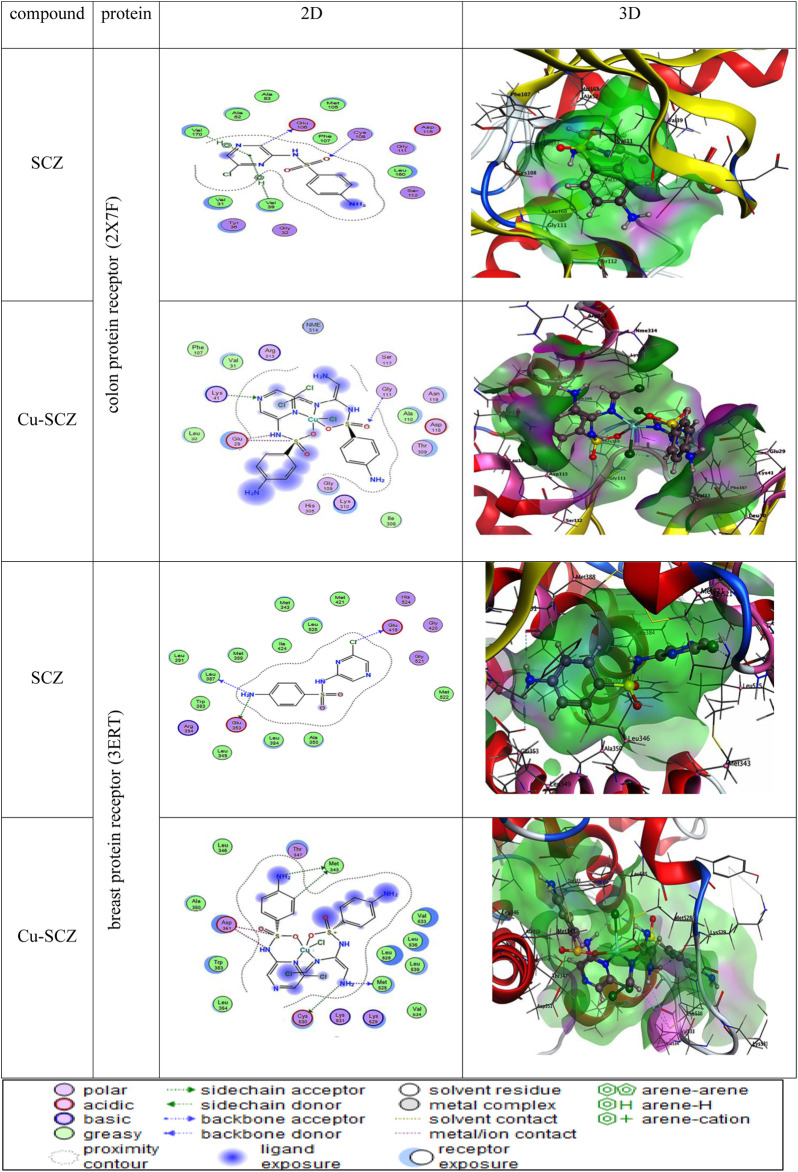

The surface maps were built over the dummy atoms as receptors to provide a better view of the molecular surface that was expressed in purple color for H-bonding, green for hydrophobic sites, and blue for polarity. The ligand and metal complex exhibited high occupancy inside the groove surface for both investigated proteins. Thus, good inhibitory activity is predicted for those compounds.

#### Cytotoxicity Results

There are several human cancer cell lines derived from different cancer types that have been commonly used to evaluate the anticancer properties of potential drugs. Among those types, we selected the breast cancer cell line (MCF-7), which is a good candidate particularly for estrogen receptor (ER)–positive breast cancer cell experiments (Sweeney et al., 2012; Comşa et al., 2015), and CaCo-2, which expresses normal enterocytic phenotypes (Hirata et al., 1993).

In vitro cytotoxicity of SCZ and the metal complexes was examined with the two cancer cell lines, human breast cancer (MCF-7) and human colon cancer (CaCo-2). The obtained results for the free drug and metal complexes are listed in Table 6 as the inhibitory concentration (IC_50_) for each cell line and SD values. Initially, the results matched the docking prediction, revealing that the Cu–SCZ complex has the highest activity against the human colon cancer cell line (CaCo-2) with IC_50_ = 23.84 µg/ml. This is more effective than that reported for Ru(III) complexes of sulfadimidine against colon cell lines (Refat et al., 2016). There are some features of copper complexes that can contribute to them forming more functional metal drugs for cancer treatments (Martin et al., 2018). For example, the associated compounds can modulate the properties of the metal ions and enhance solubility in extracellular fluids (Jungwirth et al., 2011). Moreover, they can also balance their lipophilic–hydrophilic properties to traverse the two layers’ lipid membrane (Santini et al., 2013).

**TABLE 6:** Cytotoxic activity of SCZ and its metal complexes against human tumor cells and SD values.

Compound	In vitro cytotoxicity IC_50_ (µg/ml)^1^/SD			
	Breast cell line (MCF-7)	SD	Colon cell line (CaCo-2)	SD
SCZ ligand	215.24	±0.67	97.6	±0.45
[Cu(SCZ)_2_Cl]Cl	86.2	±0.64	23.84	±0.33
[Zn(SCZ)_2_]Cl_2_	111.91	±0.36	198.44	±0.25
[Ni(SCZ)_2_Cl_2_]	45.62	±0.28	106.87	±0.34
[Co(SCZ)_2_ClOH_2_]Cl	54.23	±0.52	190.1	±0.30
[Fe(SCZ)_2_Cl_2_]	284.25	±0.31	362.9	±0.41

1 IC_50_ (mg/ml): 1–10 (very strong), 11–20 (strong), 21–50 (moderate), 51–100 (weak), and above 100 (non-cytotoxic).

In the breast cell line (MCF-7), the Ni(II) complex could also inhibit the growth of breast cancer lines (MCF-7) from 45.62 µg/ml to 50%. This effectiveness likely derives from the practical roles of nickel ions in cellular functions and their abundance in the human body (Deo et al., 2016).

Other tested compounds showed varied results, and those with values between 51 and 100 µg/ml were weakly cytotoxic, while those above 100 µg/ml were non-cytotoxic.

## Conclusion

The new metal-based drugs, Cu(II), Co(II), Zn(II), Ni(II), and Fe(II), of sulfaclozine complexes were synthesized, and their structures were affirmed by various analytical approaches. The molar ratio method indicated that the ratio of the metal to the ligand was 1:2. Moreover, spectroscopic data from IR spectroscopy showed that SCZ is a bidentate ligand coordinated by one oxygen atom of the SO_2_ group and the pyrazine nitrogen atom. Moreover, the absorption results revealed that the Fe(II), Co(II), and Ni(II) metal complexes have an octahedral structure. The solid EPR spectrum showed a trigonal bipyramidal geometry for the Cu(II) complex. The thermal decomposition assignments agreed with the suggested structure of the obtained complexes. The optimized geometries were match the experimental-suggested structures. The energy gap, E_g_, values for the complexes were lower than that of the ligand, meaning that the complexes are more reactive. The low computed hardness parameter of Cu(II) revealed strong bio-reactivity. The DNA K_b_ values were presented in descending order, Cu(II) > N(II) = Fe(II) > Co(II) > Zn(II), and were greater than the binding constant for the free ligand (6.67 × 10^5^ M^-1^). The computed free binding energy for the two proteins, breast cancer receptor protein and colon cancer receptor protein, illustrated the lowest negative score for the Cu(II) and Ni(II) complexes. The experimental cytotoxicity results presented a moderated anticancer strength of the Cu–SCZ compound. Finally, we suggest as a future perspective to study the activity of these complexes or other metal complexes by external inducements such as light or oxidizing materials.

## Data Availability

The original contributions presented in the study are included in the article/Supplementary Material, and further inquiries can be directed to the corresponding author.
