# Supplementary material for: Practical and Computational Studies of Bivalence Metal Complexes of Sulfaclozine and Biological Studies
Source: Front Chem. 2021 Jun 15;9:644691. doi: 10.3389/fchem.2021.644691 (PMC8239243; doi:10.3389/fchem.2021.644691)
Supplement: Supplementary file 1 [file DataSheet1.docx]

**Anticancer Screening of Synthesized Sulfaclozine Metal Complexes; Characterization, Molecular docking and DNA binding Study**.

Abeer A. Sharfalddin^a^, Abdul-Hamid Emwas^b^, Mariusz Jaremko^c^, Mostafa A. Hussien*^a,d^

a. Department of Chemistry, Faculty of Science, King Abdulaziz University, P.O. Box 80203 Jeddah 21589, Saudi Arabia

b. King Abdullah University of Science and Technology (KAUST), Thuwal, 23955-6900, Saudi Arabia

c. King Abdullah University of Science and Technology (KAUST), Biological and Environmental Science and Engineering (BESE), Thuwal, 23955-6900, Saudi Arabia

d. Department of Chemistry, Faculty of Science, Port Said University, Port Said, 42521, Egypt

Corresponding author:

Mostafa A. Hussien*

**Figure 1S.** Molar ratio method curves of the SCZ metal complexes

**Figure 2S**. FTIR vibrational spectra of SCZ and Cu(II), Co(II), Ni(II), Fe(II) and Zn(II) complexes.

**Table 1S.** Vibration bands (cm^-1^) in the spectra of SCZ and its metal complexes.

| M-O | M-N | S-N | C=N (aromatic) | SO_2(asy-sy)_ | NH_2_ | Metal complex |
| --- | --- | --- | --- | --- | --- | --- |
| - | - | 954 | 1588 | 1345-1149 | 3295-2966 | SCZ |
| 726 | 424 | 953 | 1584 | 1348-0 | 3291-2965 | [Cu(SCZ)_2_Cl] Cl |
| 717 | 421 | 955 | 1583 | 1348-0 | 3294-2966 | [Co(SCZ)_2_Cl OH_2_] Cl |
| 719 | 421 | 954 | 1584 | 1350-0 | 3291-2966 | [Ni(SCZ)_2_Cl_2_] |
| 757 | 419 | 955 | 1582 | 1350-1137 | 3295-2964 | [Fe(SCZ)_2_Cl_2_] |
| 726 | 422 | 955 | 1584 | 1348-0 | 3295-2966 | [Zn(SCZ)_2_] Cl_2_ |

**Figure 3S.** Absorbance spectra of SCZ ligand and metal complexes.

**Table 2S.** Horowtiz-Metzger (HM) and Coats-Redfern (CR) plots for the metal complexes.

| Coats-Redfern (CR) | Horowtiz-Metzger (HM) | Temp. range | Complex |
| --- | --- | --- | --- |
|  |  | 115-131 | [Fe(SCZ)_2_Cl_2_].3H_2_O |
|  |  | 157-177 |  |
|  |  | 302.33-340 |  |
|  |  | 625-660 |  |
|  |  | 163-172 | [Cu(SCZ)_2_Cl]Cl.H_2_O |
|  |  | 215-246 |  |
|  |  | 150-160 | [Co(SCZ)_2_Cl H_2_O]Cl.H_2_O |
|  |  | 205-240 |  |
|  |  | 217-260 | [Zn(SCZ)_2_] Cl_2_ |
|  |  | 79-94 | [Ni(SCZ)_2_Cl_2_].2H_2_O |
|  |  | 199-234 |  |

**Table** **3S.** Thermodynamic data for the decomposition stages for the metal complexes

| Complex | Temp. range C | Method | Parameter | | | | | Correlation  coefficient  (r) |
| --- | --- | --- | --- | --- | --- | --- | --- | --- |
|  |  |  | Ea  (kJmol^−1^) | A  (s^−1^) | -ΔS  (J mol^-1^ K^-1^) | ΔH  (kJmol^−1^) | ΔG  (kJ mol^−1^) |  |
| [Co(SCZ)_2_Cl OH_2_] Cl | 150-160 | CR  HM  Average | 4.81×10^5^  4.88×10^5^  2.65×10^5^ | 2.84×10^3^  4.78×10^3^  3.81×10^3^ | 1.82×10^2^  1.77×10^2^  1.80×10^2^ | 4.77×10^5^  4.52×10^4^  2.61×10^5^ | 5.55×10^5^  1.21×10^5^  3.38×10^5^ | 0.9833  0.9826  0.9830 |
|  | 205-240 | CR  HM  Average | 1.80×10^4^  5.15×10^4^  3.47×10^4^ | 3.42×10^2^  1.20×10^3^  7.69×10^2^ | 2.01×10^2^  1.90×10^2^  1.95×10^2^ | 1.39×10^4^  4.74×10^4^  3.06×10^4^ | 1.13×10^5^  1.41×10^5^  1.27×10^5^ | 0.9614  0.9647  0.9631 |
| [Cu(SCZ)_2_Cl] Cl | 163-172 | CR  HM  Average | 4.07×10^5^  4.15×10^4^  2.24×10^4^ | 2.19×10^4^  3.51×10^2^  1.11×10^4^ | 1.65×10^2^  1.99×10^2^  1.82×10^2^ | 4.03×10^5^  3.78×10^4^  2.21×10^5^ | 3.76×10^5^  1.26×10^5^  3.01×10^5^ | 0.9694  0.9812  0.9753 |
|  | 215-246 | CR  HM  Average | 2.81×10^5^  2.86×10^4^  1.55×10^4^ | 1.81×10^3^  2.12×10  4.84×10^3^ | 1.73×10^2^  2.43×10^2^  2.08×10^2^ | 2.77×10^5^  2.44×10^4^  1.51×10^5^ | 3.46×10^5^  1.47×10^5^  2.55×10^5^ | 0.9650  0.9323  0.9486 |
| [Zn(SCZ)_2_] Cl_2_ | 217-260 | CR  HM  Average | 6.31×10^3^  2.56×10^4^  1.59×10^4^ | 1.24×10^2^  8.32×10  4.22×10 | 2.86×10^2^  2.51×10^2^  2.68×10^2^ | 2.07×10^3^  2.14×10^4^  1.17×10^4^ | 1.48×10^5^  1.49×10^5^  1.48×10^5^ | 0.9141  0.9795  0.9468 |
| [Fe(SCZ)_2_Cl_2_].3H_2_O | 115-131 | CR  HM  Average | 1.67×10^5^  1.72×10^4^  9.44×10^4^ | 4.95×10^3^  4.11×10  2.47×10^3^ | 1.77×10^2^  2.55×10^2^  2.16×10^2^ | 1.64×10^5^  1.39×10^4^  8.89×10^4^ | 2.34×10^5^  1.15×10^5^  1.74×10^5^ | 0.9557  0.9737  0.9647 |
|  | 157-177 | CR  HM  Average | 3.24×10^5^  3.19×10^4^  1.78×10^4^ | 1.39×10^4^  2.09×10  6.96×10^3^ | 1.69×10^2^  2.23×10^2^  1.96×10^2^ | 3.95×10^5^  2.82×10^4^  1.73×10^5^ | 3.98×10^5^  1.24×10^5^  2.60×10^5^ | 0.98222  0.97788  0.9800 |
|  | 302.33-340 | CR  HM  Average | 2.44×10^4^  6.52×10^3^  1.55×10^4^ | 5.06×10^2^  2.46×10^3^  2.54×10^2^ | 1.98×10^2^  2.99×10^2^  2.48×10^2^ | 2.02×10^4^  2.34×10^3^  1.36×10^4^ | 1.22×10^5^  1.53×10^5^  1.36×10^5^ | 0.9797  0.9896  0.9846 |
|  | 625-660 | CR  HM  Average | 2.84×10^4^  7.13×10^3^  1.78×10^4^ | 5.79×10^2^  3.10×10^3^  2.90×10^2^ | 1.96×10^2^  2.97×10^2^  2.47×10^2^ | 2.42×10^4^  2.94×10^3^  1.36×10^4^ | 1.23×10^5^  1.53×10^5^  1.38×10^5^ | 0.9881  0.9584  0.9733 |
| [Ni(SCZ)_2_Cl_2_].2H_2_O | 79-94 | CR  HM  Average | 3.11×10^5^  3.19×10^4^  1.7110^5^ | 1.56×10^4^  2.10×10^2^  7.90×10^3^ | 1.66×10^2^  2.02×10^2^  1.83×10^2^ | 3.08×10^5^  2.89×10^4^  1.68×10^5^ | 3.68×10^5^  1.03×10^5^  2.35×10^5^ | 0.9260  0.9873  0.9566 |
|  | 199-234 | CR  HM  Average | 2.26×10^5^  2.36×10^4^  1.25×10^5^ | 6.92×10^3^  6.35×10^1^  3.46×10^3^ | 1.76×10^2^  2.53×10^2^  2.14×10^2^ | 2.22×10^5^  1.95×10^4^  1.21×10^5^ | 3.08×10^5^  1.44×10^5^  2.26×10^5^ | 0.9907  0.9823  0.9865 |

**Table 4S.** Important optimized bond lengths (Å) and bond angles (°) of CMZ and the metal complexes.

|  | SCZ ligand | Cu-SCZ | Co-SCZ | Ni-SCZ | Fe-SCZ | Zn-(SCZ) |
| --- | --- | --- | --- | --- | --- | --- |
| Bond length | | | | | | |
| C12-N11 | 1.40 | 1.39 | 1.42 | 1.39 | 1.42 | 1.39 |
| N11-S7 | 1.70 | 1.83 | 1.88 | 1.81 | 1.86 | 1.86 |
| S7-O10 | 1.45 | 1.65 | 1.68 | 1.69 | 1.66 | 1.66 |
| S7-O9 | 1.46 | 1.62 | 1.61 | 1.61 | 1.62 | 1.61 |
| S7-C6 | 1.78 | 1.83 | 1.82 | 1.82 | 1.83 | 1.80 |
| N17-C12 | 1.34 | 1.37 | 1.38 | 1.38 | 1.39 | 1.37 |
| M-O10 | - | 2 | 2 | 1.94 | 2 | 2 |
| M-N17 | - | 2.1 | 2 | 2.1 | 2.1 | 2.1 |
| M-Cl | - | 2.3 | 2.3 | 2.3 | 2.4 | - |
| M-OH_2_ |  | - | 1.95 | - | - | - |
| Bond angle | | | | | | |
| C12-N11-S7 | 126.4 | 120.3 | 109.3 | 120 | 110.2 | 126 |
| C6-S7-O10 | 108.9 | 111.3 | 109 | 108.8 | 109.2 | 110.6 |
| C6-S7-O9 | 108.2 | 111.8 | 112.4 | 113 | 111.2 | 114.6 |
| N17-C12-N11 | 114.8 | 117.7 | 119.2 | 120.6 | 121.2 | 121.9 |
| O10-M-N17 | - | 92.6 | 84 | 94.5 | 89.2 | 95.8 |
| O38-M-Cl28 | - | 91.9 | 95.5 | 84.8 | 95 | - |
| O10-M-Cl | - | 173.1 | 173.6 | 85 | 96.9 | - |
| N17-M-N45 | - | 163.5 | 170.1 | 97.2 | 101.2 | 130.2 |

**Table 5S.** Gaussian parameters using DFT/B3LYP for SCZ and prepared compounds

| Reactivity descriptor | SCZ | Cu-SCZ | Co-SCZ | Ni-SCZ | Fe-SCZ | Zn-(SCZ) |
| --- | --- | --- | --- | --- | --- | --- |
| E_g_(eV) | 3.97 | 1.76 | 3.01 | 1.85 | 3 | 2.73 |
| HOMO (eV) | -6.75 | -5.60 | -8.91 | -11.68 | -5.96 | -11.15 |
| LUMO (eV) | -2.78 | -3.84 | -5.90 | -9.83 | -2.96 | -8.42 |
| η (eV) | 1.99 | 0.67 | 1.51 | 0.93 | 1.50 | 1.37 |
| S(1/eV) | 0.99 | 0.32 | 0.75 | 0.46 | 0.75 | 0.68 |
| μ(eV) | -4.77 | -1.59 | -7.41 | -10.76 | -4.46 | -9.79 |
| ω(eV) | 5.72 | 0.80 | 3.70 | 5.83 | 2.23 | 4.89 |

**Table 6S.** Binding parameters for SCZ drug and its complexes from the spectrophotometric analysis.

| Complex | K_b_ (M^-1^) | ΔG (kJ mol) | Chromism (%) | λ_max_ Free (nm) | λ_max_ Bound (nm) | Type of chromism |
| --- | --- | --- | --- | --- | --- | --- |
| SCZ | 4.5 ×10^5^ | -3.15×10^4^ | 2.28 | 263 | 257 blue shift | Hyperchromic |
| [Cu(SCZ)_2_Cl] Cl | 9×10^5^ | -3.25×10^4^ | 2.67 | 262 | 255 blue shift | hypochromic |
| [Co(SCZ)_2_Cl OH_2_] Cl | 5×10^5^ | -3.20×10^4^ | 5.36 | 280 | 265 blue shift | hypochromic |
| [Ni(SCZ)_2_Cl_2_] | 6.67×10^5^ | -3.25×10^4^ | 5.19 | 270 | 256 blue shift | hypochromic |
| [Fe(SCZ)_2_Cl_2_] | 6.67×10^5^ | -3.16×10^4^ | 3 | 266 | 258 blue shift | hypochromic |
| [Zn(SCZ)_2_] Cl_2_ | 6.6×10^5^ | -3.32×10^4^ | 2.65 | 264 | 257blue shift | hypochromic |

**Table 8S**. Energy scores (Kcal mol^-1^) calculation for SCZ ligand and divalent complexes with colon proteins 2X7F and 4F9M

| Name |  | S | Rmsd-refine | E-conf | E-place | E-score1 | E-refine | E-score2 |
| --- | --- | --- | --- | --- | --- | --- | --- | --- |
| SCZ Ligand | colon protein (2X7F) | -5.67657 | 1.391323 | -38.6837 | -78.4848 | -8.32963 | -23.4044 | -5.67657 |
| [Cu(SCZ)_2_Cl]Cl |  | -6.67613 | 2.674901 | 41.1056 | -60.2885 | -11.2292 | -30.7477 | -6.36569 |
| [Zn(SCZ)_2_ ]Cl |  | -6.36569 | 1.714471 | -640.054 | -81.1297 | -10.9456 | -34.3364 | -6.67613 |
| [Ni(SCZ)_2_Cl_2_] |  | -5.68732 | 2.10175 | 15.49966 | -58.7423 | -10.3486 | -25.0419 | -5.68732 |
| [Co(SCZ)_2_ClOH_2_]Cl |  | -6.39108 | 2.768533 | -164.495 | -81.2272 | -11.2609 | -34.5824 | -6.39108 |
| [Fe(SCZ)_2_Cl_2_] |  | -6.39108 | 1.62747 | -295.291 | -54.6989 | -11.0397 | -32.7297 | -6.45728 |

| Name |  | S | Rmsd-refine | E-conf | E-place | E-score1 | E-refine | E-score2 |
| --- | --- | --- | --- | --- | --- | --- | --- | --- |
| SCZ Ligand | colon protein (4FM9) | -6.55138 | 0.726084 | -19.4681 | -68.6865 | -9.5663 | -23.0482 | -6.55138 |
| [Cu(SCZ)_2_Cl]Cl |  | -7.24939 | 1.968041 | -121.83 | -94.3268 | -10.0618 | -23.8691 | -7.24939 |
| [Zn(SCZ)_2_ ]Cl |  | -7.2411 | 3.451702 | -398.736 | -75.5896 | -10.3528 | -31.8769 | -7.2411 |
| [Ni(SCZ)_2_Cl_2_] |  | -6.88243 | 1.150235 | -59.7293 | -89.1016 | -10.3708 | 21.92744 | -6.88243 |
| [Co(SCZ)_2_ClOH_2_]Cl |  | -6.71789 | 2.501064 | -246.793 | -55.2867 | -10.8246 | -16.3914 | -6.71789 |
| [Fe(SCZ)_2_Cl_2_] |  | -6.80609 | 1.248954 | -146.58 | -83.7791 | -11.2369 | -19.4578 | -6.80609 |

**Table 9S.** Energy scores (Kcal mol^-1^) calculation for SCZ ligand and divalent complexes with breast proteins, 3ERT and 1H7K

| Name |  | S | Rmsd-refine | E-conf | E-place | E-score1 | E-refine | E-score2 |
| --- | --- | --- | --- | --- | --- | --- | --- | --- |
| SCZ Ligand | breast protein (3ERT) | -6.02021 | 1.250451 | -37.5798 | -40.7593 | -8.25964 | -28.4376 | -6.32021 |
| [Cu(SCZ)_2_Cl]Cl |  | -6.5352 | 1.518553 | -119.352 | -9.49298 | -9.17079 | -34.6858 | -6.5352 |
| [Zn(SCZ)_2_ ]Cl |  | -6.01604 | 2.561127 | -642.22 | -32.2478 | -11.2108 | -33.3199 | -6.01604 |
| [Ni(SCZ)_2_Cl_2_] |  | -6.0157 | 1.896875 | 6.161269 | -31.901 | -9.90892 | -34.8761 | -6.0157 |
| [Co(SCZ)_2_ClOH_2_]Cl |  | -6.18126 | 1.670435 | -164.519 | -31.6865 | -8.94235 | -33.0142 | -6.18126 |
| [Fe(SCZ)_2_Cl_2_] |  | -6.24734 | 3.013468 | -288.61 | -27.7413 | -10.7803 | -28.6077 | -6.24734 |

| Name |  | S | Rmsd-refine | E-conf | E-place | E-score1 | E-refine | E-score2 |
| --- | --- | --- | --- | --- | --- | --- | --- | --- |
| SCZ Ligand | breast protein (1hk7) | -5.49007 | 1.302449 | -38.3002 | -32.9096 | -5.98494 | -25.461 | -5.49007 |
| [Cu(SCZ)_2_Cl]Cl |  | -7.73247 | 2.632134 | -115.452 | -6.28986 | -8.55733 | -27.6291 | -7.73247 |
| [Zn(SCZ)_2_ ]Cl |  | -7.10101 | 2.18352 | -376.78 | -20.9108 | -7.42893 | -27.6352 | -7.10101 |
| [Ni(SCZ)_2_Cl_2_] |  | -6.80484 | 2.031628 | -158.137 | -18.1228 | -7.94991 | -28.9851 | -6.80484 |
| [Co(SCZ)_2_ClOH_2_]Cl |  | -6.22617 | 2.229991 | -208.21 | -15.1401 | -7.74724 | -35.9834 | -6.22617 |
| [Fe(SCZ)_2_Cl_2_] |  | -6.55553 | 2.164933 | -154.379 | -35.3824 | -7.67092 | -35.9025 | -7.55553 |
